# The neurobiology and treatment of first-episode schizophrenia

**DOI:** 10.1038/mp.2014.66

**Published:** 2014-07-22

**Authors:** R S Kahn, I E Sommer

**Affiliations:** 1 Department of Psychiatry, Brain Center Rudolf Magnus, University Medical Centre Utrecht, Utrecht, The Netherlands

## Abstract

It is evident that once psychosis is present in patients with schizophrenia, the underlying biological process of the illness has already been ongoing for many years. At the time of diagnosis, patients with schizophrenia show decreased mean intracranial volume (ICV) as compared with healthy subjects. Since ICV is driven by brain growth, which reaches its maximum size at approximately 13 years of age, this finding suggests that brain development in patients with schizophrenia is stunted before that age. The smaller brain volume is expressed as decrements in both grey and white matter. After diagnosis, it is mainly the grey matter loss that progresses over time whereas white matter deficits are stable or may even improve over the course of the illness. To understand the possible causes of the brain changes in the first phase of schizophrenia, evidence from treatment studies, postmortem and neuroimaging investigations together with animal experiments needs to be incorporated. These data suggest that the pathophysiology of schizophrenia is multifactorial. Increased striatal dopamine synthesis is already evident before the time of diagnosis, starting during the at-risk mental state, and increases during the onset of frank psychosis. Cognitive impairment and negative symptoms may, in turn, result from other abnormalities, such as NMDA receptor hypofunction and low-grade inflammation of the brain. The latter two dysfunctions probably antedate increased dopamine synthesis by many years, reflecting the much earlier presence of cognitive and social dysfunction. Although correction of the hyperdopaminergic state with antipsychotic agents is generally effective in patients with a first-episode psychosis, the effects of treatments to correct NMDA receptor hypofunction or low-grade inflammation are (so far) rather modest at best. Improved efficacy of these interventions can be expected when they are applied at the onset of cognitive and social dysfunction, rather than at the onset of psychosis.

## Introduction

When does schizophrenia first manifest itself? Is it at the onset of the first psychosis? Is it at the first signs of psychosis, as in the group of patients referred to as ‘at-risk mental state' (ARMS)? Or is it even earlier, and not primarily associated with psychosis, but with cognitive decline? This question is not only essential to address the biology of first-episode schizophrenia, but it is at the core of the schizophrenia concept itself.

We have argued that the first signs of schizophrenia already occur in early puberty with a (relative) decline in cognitive dysfunction. It is not until many years later, when psychotic symptoms occur during the ARMS, or more pronounced during the first psychosis itself, that the diagnosis becomes obvious.^1^ Thus, we would argue that first-episode schizophrenia is a misnomer, as the core of the illness, that is, the cognitive decline, may not be episodic. Nevertheless, almost all studies into the biology of the first stages of schizophrenia have focused on the psychotic symptoms to define the onset of the illness.

## Brain changes at the time of first psychosis

It is evident that once psychosis is present in patients with schizophrenia, the underlying biological process of the illness has already been ongoing for many years. This conclusion can be based on the multitude of neuroimaging studies that we recently reviewed in a meta-analysis of over 18 000 subjects, including 771 medication-naive, recent onset patients.^2^ These data show a slight, but significant, decrease in intracranial volume in patients with schizophrenia (effect size−0.2), in chronic and recent onset, medication-naive patients. Intracranial volume is driven by brain growth, as it is the enlarging brain that determines the expansion of the skull.^3,4^ The growing brain reaches its maximum size at approximately 13 years of age.^5^ Therefore, brain development must be stunted in patients with schizophrenia before that time. From the same meta-analysis, it can be gleaned that there must be additional brain loss, or continued abnormal development, after the age of 13: total brain volume in never-treated patients is decreased to a larger degree (effect size−0.4) than is intracranial volume and this is due to decreases in both white and grey matter.^2^ Importantly, while grey matter loss is larger in chronic than in medication-naive patients, white matter volume is decreased to a similar extent in both groups. Indeed, longitudinal studies indicate that loss of white matter volume, while present at psychosis onset, does not progress further after psychosis has emerged.^6^ This is consistent with the finding in twin studies that decreased white matter volume in schizophrenia may be related more to the genetic risk to develop the illness than to the effects of illness itself.^7^ In contrast, grey matter volume loss (mainly expressed as reductions in cortical thickness) progresses further after the onset of psychosis, and is related to outcome,^8^ cannabis smoking,^9^ medication use^10,11^ and psychotic relapses.^12^ Thus, although some of the brain abnormalities in schizophrenia worsen after the onset of psychosis, abnormal development of the brain must have been ongoing for many years before the first psychosis—expressed, as it is, in decreased intracranial volume and even larger decreases in white and grey matter.

What is the nature of the white and grey matter changes that are present at the onset of the first psychosis? Using tract-based analysis of white matter fibres in medication-naive schizophrenia patients we, and others, have found differences in the uncinate and arcuate fasciculi, suggestive of axonal or glial damage and/or increased free water concentrations.^13,14^ In unmedicated first-episode psychosis (FEP) patients reduced fractional anisotropy, a measure reflecting white matter fibre density and myelination, is related to cognitive dysfunction.^15^ Pronounced fractional anisotropy reductions in medication-naive FEP patients appear to be predictive of poor response to subsequent antipsychotic treatment.^14^ While white matter decreases are not evenly dispersed throughout the brain, but instead are most pronounced in association fibres, such as the uncinate and arcuate fasciculi, changes in the grey matter are not uniformly distributed throughout the brain either.^8^ Most pronounced grey matter decreases in FEP patients are found in frontal and temporal areas, including the insula, superior temporal gyrus and the anterior cingulate gyrus.^8,16^ As indicated, following the FEP, most (but not all) longitudinal studies suggest that grey matter loss continues, which is most prominent in frontal and temporal areas, and results from cortical thinning (and not surface shrinkage) and is related to clinical and cognitive outcome.^9,17, 18, 19, 20^ Only few studies have investigated white matter changes over time after the FEP.^21^ Two recent studies showed contrasting results with one demonstrating improvement of white matter deficits in FEP patients after antipsychotic treatment^14^ and the other showing worsening of these abnormalities.^22^ On postmortem examination, decreases in white matter are associated with a reduction in oligodendrocytes in the superior frontal cortex^23^ and in the bilateral hippocampus,^24^ suggesting dysfunction of oligodendrocytes to underlie white matter deficits in schizophrenia.

## Brain changes before the onset of the first psychosis

The ARMS is a prodromal phase of schizophrenia characterized by cognitive impairments,^25^ mood alterations,^26^ anxiety,^27^ attenuated psychotic symptoms and a decline in social and occupational functioning.^28^ Although the concept has been useful in understanding the development of schizophrenia, only a small percentage of patients with these symptoms eventually go on to develop the illness—and this percentage further declines as the number of studies increases.^29^

A recent review on neurobiological changes in ARMS subjects suggests that volumes of frontal and temporal areas are decreased in a similar fashion—but to a lesser extent—as observed in schizophrenia.^30^ Longitudinal studies are scarce, but those available suggest that grey matter deficits present in those subjects that go on to develop schizophrenia, worsen over time and are found mainly in fronto-temporal areas.^31,32^ Progressive reduction in the integrity of frontal white matter has also been reported in ARMS subjects who go on to develop schizophrenia.^33^ However, studies in the ARMS period are limited by the fact that the subjects studied are selected on the basis of the presence of mild and incomplete symptoms of psychosis and that outcome, that is, conversion, is defined by psychosis as well. It has been argued that a focus on cognitive and negative symptoms in these ARMS subjects may be needed to understand the developmental biology of schizophrenia.^27^ Indeed, baseline cognitive functioning in ARMS subjects is an adequate predictor of poor outcome, regardless of transition to psychosis.^34, 35, 36^

## Possible causes and effects of the brain changes

To understand the possible causes of the brain changes in the first phase of schizophrenia, evidence from treatment studies, postmortem and neuroimaging investigations together with animal experiments needs to be integrated. These studies suggest that schizophrenia is related to at least three interacting pathophysiological mechanisms: dopaminergic dysregulation, disturbed glutamatergic neurotransmission and increased proinflammatory status of the brain. These processes interact with each other and most likely have causal interrelationships.

### Dopamine dysregulation

Since the discovery of the antipsychotic properties of chlorpromazine in the 1950s, increased dopamine (DA) turnover in the striatum has received much attention as an underlying mechanism of schizophrenia. Although initial studies focused on the postsynaptic DA receptor, more recent positron emission tomogrpahy (PET) studies, using (18)F-DOPA as a tracer, show that the major locus of dopaminergic dysfunction is presynaptic rather than postsynaptic in nature, characterized by elevated DA synthesis and release capacity. Increased (18)F-DOPA binding capacity is already present during the ARMS period and is found to be predictive of the further development into full clinical psychosis^37, 38, 39^ (see Table 1A for an overview of DA deviations in ARMS subjects). In medication-naive schizophrenia patients who experience an FEP, increased striatal DA synthesis is a rather consistent finding (an overview is provided in Table 1B). Although increased striatal DA synthesis may be the final common pathway to psychotic symptoms,^40,41^ its relation to cognitive symptoms is less clear. In a mouse model, increased postsynaptic striatal DA receptors could evoke cognitive dysfunction in several domains,^42^ but this has not been tested directly in humans.

### N-methyl-D-aspartate receptor hypofunction

In fact, some of the cognitive dysfunction in schizophrenia may be related to a different neurotransmitter complex, the N-methyl-D-aspartate receptor (NMDAr)/glutamate system.^43^ It has been hypothesized that the NMDAr, situated between the primary and secondary glutamatergic cortical neurons, constitutes the main deficit underlying schizophrenia. Poor function of the NMDAr, in turn, renders the gamma-amino-buteric-acid (GABA)-ergic interneuron less effective. This loss of GABA-ergic firing provides insufficient inhibition of the secondary glutamatergic neurons, allowing them to fire more often but with less synchrony, directly causing the excessive firing of DA neurons in the mesolimbic pathway.^44^ This hypothesis is based on studies using NMDAr antagonists, such as ketamine and phencyclidine, which were found to induce the full range of schizophrenia symptoms, including psychosis, negative symptoms, and also cognitive dysfunction.^45^ Furthermore, patients with an autoimmune encephalitis producing antibodies against the NMDAr can have a clinical picture that is indistinguishable from schizophrenia.^46^ Finally, many of the well-known risk genes, such as DISC-1, dysbindin, SHANK and NRG-1,^47,48^ but also *de novo* mutations^49^ associated with schizophrenia influence glutamatergic neurotransmission.

During brain development the NMDAr has a crucial role in brain maturation by means of synaptic plasticity, which forms the basis for adequate development of higher cognitive functions, such as learning and memory (see Wang *et al.*^50^). NMDAr is a hetero-tetrameric structure with one obligate NR1 and two variable NR2 subunits, determining its biophysical and pharmacological properties. During brain development, the subunit composition of this receptor undergoes a switch, in which some subunits are replaced by structurally different ones. The mature receptor composition has different physiological properties, rendering the receptor more suitable for optimal timing of firing, thereby enabling the swift integration of environmental stimuli. The timing of receptor switches differs per brain region, and may coincide with ‘risk windows' for schizophrenia, that is, developmental phases when the individual is particularly vulnerable to environmental influences such as hypoxia, birth stress, infection or inflammation, drug abuse or social isolation.^51, 52, 53^ During pregnancy, fetal NMDAr levels are increased, rendering the infants' brain vulnerable to insults.^54^ It is conceivable, although largely hypothetical, that environmental risk factors for schizophrenia affect the brain by means of delaying or preventing adequate NMDAr switching in specific brain areas, and an incomplete receptor switch could be related to the onset of cognitive decline in the earliest phases of the illness. Imperfect expression of the mature NMDAr subunit profile is likely to impair the process of long-term depression and potentiation, by which frequently-used connections are strengthened and rarely-used connections are weakened.^50^ At early adolescence, pruning will eliminate the weak connections. When a lack of long-term depression and potentiation has resulted in a failure to differentiate the frequently-used from the rarely-used connections, pruning may become a random process, eliminating important as well as less-relevant connections.^55^

Downstream from the glutamatergic neurons, decreased functioning of the NMDAr leads to hypofunction of the inhibitory GABA-ergic interneurons. Decreased functioning of these fast-spiking GABA-ergic interneurons hampers synchronisation of neuronal firing of the pyramidal neurons. Diminished synchronized neuronal activity leads—again—to impaired cognitive processing.^56^ Postmortem studies consistently demonstrate that a subpopulation of the GABA-ergic interneurons, the parvalbumin-containing chandelier cells, is decreased in patients with schizophrenia (for a review see Curley *et al.*^57^). Enzymes related to GABA-ergic neurotransmission, such as glutamic acid decarboxylase (GAD)67 and GABA transporter (GAT)1, are consistently reported to be decreased in patients with schizophrenia.^57^ A large postmortem study involving 240 controls of all age categories and 31 patients with schizophrenia observed that development and maturation in the prefrontal cortex and the hippocampus is characterized by progressive switches in expression from GAD25 to GAD67 and from NKCC1 to KCC2. The former switch leads to GABA synthesis, and the latter leads to switching from excitatory to inhibitory neurotransmission. In the hippocampus, GAD25/GAD67 and NKCC1/KCC2 ratios are increased in patients with schizophrenia, reflecting a potentially immature GABA physiology.^58^ This deviation was associated with the risk allele at the promoter region of the GAD-1 gene.^58^

It remains unclear whether deviations in the GABA-ergic interneurons are secondary to deficits in NMDAr-mediated signalling, or if abnormal NMDAr signalling is compensatory to GABA-ergic aberrations. Either way, hypofunction of the NMDAr and reduced neural synchrony caused by decreased function of the GABA-ergic interneurons may be the converging mechanisms underlying cognitive dysfunction, which—as indicated—starts at least 10 years before the onset of psychotic symptoms^1^ and remains relatively stable after the FEP, as a 10-year follow-up study of FEP patients showed no clear signs of deterioration as compared with healthy controls.^59^ Murine studies show that glutamatergic afferents from the hippocampus to the nucleus accumbens exert a strong excitatory effect on striatal DA neurons, influencing both activity and firing properties of the dopaminergic neurons.^60^ Thus, decreased activation of the NMDAr leads to an increase in striatal DA release and induce psychotic symptoms.^61^ This finding provides a biological explanation of the clinical and epidemiological observations that cognitive changes precede the onset of psychosis by many years.^1^

One of the few available techniques to examine the status of the NMDA/glutamate system in the human brain is the use of magnetic resonance spectroscopy (MRS). This method provides concentrations of several molecules, including glutamate, glutamine and GABA.^62^ However, glutamate as measured with MRS does not reflect intrasynaptic glutamate levels, as the MRS signal is derived from glutamate in neurons, blood vessels, white matter, and so on.^62^ When glutamate is released into the synapse it is quickly metabolized into the inert glutamine, which may be a better reflection of intrasynaptic glutamate levels and hence of NMDAr hypofunction. Indeed, Rowland *et al.*^63^ found increased glutamine as measured with MRS after infusion of ketamine in healthy subjects. Moreover, with magnetic resonance imaging scanners at a magnetic field strength lower than 4 Tesla, it is difficult to disentangle the peaks from glutamate and glutamine; most studies therefore provide a value of ‘glx', which is composed of both glutamate and glutamine. Results in schizophrenia suggest that glx concentrations are different for each stage of the illness.

Although results in ARMS subjects are not consistent, the majority of studies show increased glx,^64, 65, 66, 67, 68^ whereas a few report decreased^69,70^ or normal^71,72^ values (see Table 2A). Studies differentiating between glutamate and glutamine generally report increased levels of both molecules. In medication-naive FEP patients, studies generally report increased glx concentrations (composed of increased glutamate and increased glutamine) as compared with healthy controls,^67,73, 74, 75^ whereas in medicated FEP patients, glx levels are reported to be normal (Table 2B).^76, 77, 78, 79^ In the later phases of schizophrenia, glx values appear slightly but significantly decreased, which is the result of decreased glutamate and increased glutamine levels, leading to an increased glutamine-to-glutamate ratio.^80,81^ The decreased glx levels in chronically medicated patients are most pronounced in the frontal areas and correlate with cognitive deficits.^80^

GABA levels have been measured less extensively but the few available reports generally indicate decreased GABA levels in medicated FEP as well as in chronic patients, and these are correlated with cognitive dysfunction,^62,82,83^ but see Tayoshi *et al.*^84^ Detailed information on GABA levels in ARMS subjects and in unmedicated FEP patients is as yet unavailable.

### Increased proinflammatory status

The third mechanism that may underlie (some of the) the signs and symptoms of schizophrenia is an increased proinflammatory status of the brain, a hypothesis proposed many years ago, for example, by Stevens^85^ who observed signs of low-grade inflammation in postmortem brains of patients with schizophrenia. Interest in inflammation as a possible aetiology of schizophrenia has been bolstered by the simultaneous publication of three genome-wide association studies in 2009 providing compelling evidence for the involvement of the MHC region in the susceptibility of schizophrenia.^86, 87, 88^ MHC class I molecules could also operate through direct effects on brain development as these molecules regulate many aspects of brain development, including neurite outgrowth, synapse formation and function, homeostatic plasticity and activity-dependent synaptic refinement.^89, 90, 91^ However, epidemiological studies consistently show that the risk for schizophrenia is increased following pre and perinatal infections.^92^ Moreover, a nation-wide registry study has shown that both (familial) autoimmune disorders and a history of infection (severe enough to need hospital admittance) increase the risk to develop schizophrenia.^93^ A subset of patients initially diagnosed with schizophrenia is known to suffer from autoimmune encephalitis. A recent study demonstrated anti-NMDAr antibodies in almost 10% patients with schizophrenia as compared with 0.4% in controls,^94^ but replication of this finding is needed.

However, neuroinflammation probably has a role in a larger group of patients, not just in those who can be characterized as suffering from an autoimmune encephalitis.

The immature brain can be exposed to inflammation associated with viral or bacterial infection or as a result of sterile brain insults. Microglia are the main immuno-competent cells in the immature brain, and depending on the stimulus, molecular context and timing, these cells will acquire various phenotypes, which are critical regarding the consequences of inflammation.^95^ Acute inflammation can shift to a chronic inflammatory state and adversely affect brain development.

Support for the putatively increased activation of microglia cells is provided by two studies using 11C-PK11195 PET, reporting increased activation of microglia cells especially in the temporal lobes in patients with early-stage schizophrenia as compared with controls.^96,97^ A third PET study using another tracer (11C-DAA1106)^98^ found no differences between schizophrenia patients and controls. Specificity of both tracers for microglia activation is under discussion, however.^99^ A possible explanation for the difference is that the latter PET study included chronic patients and increased neuroinflammation may be present only in the first years of the disease. If this would be the case, then postmortem studies—usually including only chronic patients—would not be expected to find signs of increased inflammation. However, although results are inconsistent, many postmortem studies, in fact, do report increased numbers of microglia cells in activated states.^100^ Table 3 provides a summary of these findings. Only one postmortem study analysed brain tissue of patients with long and short duration of illness^101^ and, surprisingly, reported strongest indications of increased inflammation in the later stages of the illness. Postmortem literature, which mainly describes the late stages of schizophrenia, may therefore not be representative for the presence (or absence) of increased proinflammatory status of the brain in patients with an FEP. Information on a potential proinflammatory status in FEP patients can be retrieved from peripheral blood markers, which so far show that deviations in pro and antiinflammatory factors are of the same magnitude in FEP patients as in chronic patients with acute exacerbations.^102^

When microglial cells become activated, they abandon their neurotrophic functions (for example, axon guidance and the production of neurotrophins such as BDNF), which leave the neurons in suboptimal condition.^103^ In addition, activated microglia produce several neurotoxic substances, such as free radicals and proinflammatory cytokines that can damage neuronal and glial cells, leading to cognitive dysfunction and brain volume loss.^100^ Neuroinflammation and NMDAr dysfunction are interwoven in several ways. For example, activated microglial cells produce high levels of glutamate, whereas NMDAr activity is required for the expression of antioxidant enzymes,^104^ necessary to compensate the toxic effects of microglial activation. Furthermore, deviant brain development and subsequent cognitive alterations in adulthood may be mediated by cytokines, especially by IL-6 induction during infection.^105^ Activation of the IL-6/Nox2 pathway and consequent increase in superoxide production in the brain can also induce a loss of parvalbumin-containing interneurons in adulthood.^106^ The increased glutamate levels observed with MRS in the ARMS and early FEP period may thus result from activated microglial cells rather than from NMDAr hypofunction. The increased proinflammatory status can also cause or worsen hypoactivation of the NMDAr by means of altered tryptophane catabolism.^107^ During low-grade inflammation, the catabolism of tryptophane in the brain is shifted away from serotonin as an end product towards kynurenic acid, which inhibits the NMDAr at the glycine site.^108^ One postmortem study and several studies investigating cerebrospinal fluid indeed showed increased levels of kynurenic acid in patients with schizophrenia as compared with controls (reviewed by Coyle^109^). Inflammation can also be linked to DA dysregulation, as animal studies consistently show increased activity of mesolimbic DA neurons in offspring of rodents exposed to prenatal inflammatory challenges.^110^ In fact, the white matter alterations observed in the early stages of schizophrenia, before psychotic symptoms have become apparent, could reflect an increased inflammatory status of the brain.^111^

### Not all schizophrenia patients have the same pathophysiology

It is highly unlikely that the pathogenesis of all patients with schizophrenia will be uniform. More probable is that some patients will display for example pronounced NMDAr hypofunction, whereas in others this mechanism is hardly affected. Indeed, Egerton *et al.*^112^ have found that FEP patients who respond well to antipsychotic medication displayed normal glx levels in the anterior cingulate cortex, whereas those with poor response showed increased glx concentrations, indicating that in the nonresponders, other mechanisms than increased DA synthesis may have a role. Demjaha *et al.*^113^ confirmed that patients with intractable psychosis, not responding to various antipsychotic agents, lacked the typical increase in DA synthesis capacity. In a similar vein, increased proinflammatory status of the brain may be most pronounced in a specific subgroup of patients. Indeed, in 180 medication-naive FEP patients, approximately one-third showed marked increases in serum immunity markers.^44^ In parallel, a recent postmortem study indicated signs of low-grade inflammation in 40% patients with schizophrenia.^114^ For future research, it will be key to determine deviations in DA synthesis, NMDAr hypofunction and proinflammatory status of the brain on the subject level so that these mechanisms can be targeted on an individual basis. Neuroimaging techniques to visualize striatal DA synthesis, frontal glutamine levels and activation of microglial cells could unravel which underlying neurobiology is relevant in a specific patient.

## Treatment of first-episode schizophrenia

For obvious reasons, treatment of schizophrenia has focused almost exclusively on the stage when patients present with clear-cut clinical symptoms, that is, psychosis. Although an increasing number of studies are now developing treatment at the earlier stages of the illness, such as the ARMS, or focus on the alleviation of cognitive dysfunction in chronic patients, the bulk of studies still focus on the treatment of psychosis.

### Antipsychotic treatment

The best-known mechanism of action of antipsychotic medication is the correction of increased striatal DA turnover.^35^ Interestingly, more recent work in animals (Kato *et al.*)^115,116^ and cultured brain cells (Zheng *et al.*)^117^ suggest that inhibition of microglial activation may be an additional aspect of the efficacy of antipsychotics. Although we have had effective antipsychotic treatments for nearly 50 years, the application and implementation of these treatments is far from optimal. Many of the elementary questions in the treatment of schizophrenia have remained unanswered. Fortunately, first-episode patients do often respond reasonably well;^118^ the main challenge then becomes how to keep them well.^119^ Once it has been decided that antipsychotic treatment is to be initiated, the question arises on how to prioritize the currently available treatments in a rational and optimal manner. No one treatment will be adequate for all patients. Prospective, sequential studies are necessary to develop treatment algorithms for schizophrenia, but these are almost completely missing. Although every year hundreds of studies on schizophrenia are published (the register of the Cochrane Schizophrenia Group currently includes 12 000 controlled clinical trials), most of the studies focus on the question of whether a specific drug or psychotherapeutic intervention works or not. However, lacking are the mechanism-based, rational, sequential studies that address how to deal with treatment nonresponse. Although schizophrenia patients with an FEP are highly responsive to antipsychotic medication,^118^ this rapidly diminishes as episodes increase.^120^ Whether switching of antipsychotics is helpful in such patients has hardly been studied, although several large trials are currently under way (OPTiMiSE trial and SWITCH). Agid *et al.*^121^ used an algorithm in which 244 FEP patients were randomized to risperidone or olanzapine. After 4 weeks, as much as 75% had responded to medication (82% in the olanzapine group and 66% in the risperidone group). Nonresponders were switched to the other arm. In this second trial, response rate dropped dramatically to only 17% and again significantly more patients in the olanzapine than in the risperidone group responded. This study illustrates the high response rate in FEP patients, but also shows that patients who do not respond to the first antipsychotic medication have a low probability of responding to a second antipsychotic drug. In these nonresponders, non-dopaminergic mechanisms may be important and when a first trial of antipsychotic medication has failed, treatments to correct NMDAr hypofunction, or increased proinflammatory status of the brain, are expected to be more effective.^44,112,113^

### Glutamatergic treatments

There are several routes that can potentially improve, or compensate, NMDAr hypofunction in schizophrenia. First, the availability of glycine or D-serine at the glycine site can be increased by the administration of glycine or D-serine. Some studies suggest that glycine and D-serine modestly improve positive and negative symptoms,^122^ with little or no impact on cognitive dysfunction.^123^ D-serine levels can also be increased by inhibiting its cataboliser D-amino acid oxidase (DAAO), which so far showed no efficacy on symptom severity.^124^

Modulations of AMPA receptors, which are colocalized in synapses near NMDA receptors, provide another avenue for treatment. Several compounds such as CX-516, piracetam cyclothiazide and LY404187 have been tested but so far have not shown clear benefits.^125^ A third option is modulation of the glycine transporter, for example, with sarcosine, which has demonstrated some improvement in negative and cognitive symptoms.^126^ Finally, modulation of the metabotropic glutamate receptor (mGluR) has been studied: in a phase II study, one of these substances (LY354740), was comparable in efficacy to olanzapine,^127^ but a subsequent larger trial was inconclusive.^128^

Influencing GABA-ergic interneurons—the downstream relays of the glutamatergic neurons—offers an alternative strategy. Two classes of selective GABA-ergic drugs have been proposed to enhance cognition in schizophrenia, α5-selective inverse agonists and α2/3-selective agonists. There is compelling evidence from animal models of schizophrenia that allosteric modulation of the α5 subunit of the GABA-A receptor can correct underlying deviations and lead to improvements in cognition.^129^ So far, significant improvement of cognition in patients with schizophrenia by GABA-ergic drugs has not been demonstrated, however.^130^ The disappointing results with agents targeting NMDAr-mediated or GABA-ergic signalling may not come as a surprise given the fact that dysfunction within these circuits is likely to take place far earlier than does the onset of psychosis. At the time of frank psychotic symptoms many years of NMDAr and GABA-ergic hypofunction may already have caused irreversible deficits in brain maturation and synaptic plasticity. Therefore, treatment for schizophrenia may only be truly effective during the critical developmental window, after which the brain is hard -wired.^131^ For treatment, or better prevention, of cognitive decline, it will be key to diagnose at-risk subjects much earlier than the FEP or even the ARMS stage so that glutamatergic or GABA-ergic medication can be given before the window of opportunity has closed.

### Antiinflammatory agents for the treatment of schizophrenia

The use of antiinflammatory agents to improve symptoms of schizophrenia is still in its infancy. A recent meta-analysis has shown some efficacy in schizophrenia for aspirin, n-acetylcysteine (NAC) and estrogens (the latter only in females), but not for other agents with antiinflammatory properties, such as celecoxib, minocycline, davunetide and polyunsaturated fatty acids.^132^ Two EEG studies showed that NAC improved both multivariate phase synchronization and mismatch negativity in patients with schizophrenia.^133,134^ A trial in ARMS subjects, however, did show that polyunsaturated fatty acids significantly reduced (or delayed) transition to psychosis.^135^ A follow-up study of this RCT showed that a reduction of positive symptoms and a lower mean PANSS positive score in the polyunsaturated fatty acids group were apparent after 8 weeks, whereas the significant drop in negative symptoms and the higher mean scores in global functioning occur later at 12 weeks.^136^ More studies are needed, however, before this treatment can be considered an effective intervention.

As increased proinflammatory status may also affect the brain in an early stage of the illness, augmentation with these agents during the ARMS or FEP stages may be less effective than earlier interventions, that is, several years before psychosis starts. As the diagnosis of schizophrenia is currently based on the onset of the psychotic symptoms, irreversible damage to neurons and glia cells, reflected in brain volume loss, may already be present at the time of diagnosis (as has been argued above and has been repeatedly shown in magnetic resonance imaging studies). Thus, to treat the earliest phases of the illness, antiinflammatory agents with high numbers-needed-to-harm are the best candidates. NAC may be of particular interest, as this component targets not only a diverse array of factors including glutamatergic neurotransmission, the antioxidant glutathione, neurotrophins, apoptosis, mitochondrial function, but also the inflammatory pathways.^137^ NAC displays a benign side-effect profile and may even have some anti-addictive properties,^138^ which would make this component a valuable substance for prevention of brain volume loss, cognitive deterioration and subsequent transition to psychosis in individuals at (genetic) risk for schizophrenia.

### Non-pharmacological treatments

Among the many non-pharmacological interventions recently developed to treat patients in the ARMS and FEP period, exercise interventions, such as aerobic interval training, seem especially appealing. The beneficial effects of exercise on mood and self-esteem have long been acknowledged^139^ and we recently showed that psychotic and negative symptoms are also reduced by exercise interventions as compared with creative therapy.^140^ Interestingly, physical exercise is known to affect gene expression in an antiinflammatory pathway, including the downregulation of monocyte TNF, TLR4 and CD36 genes.^141^ In sedentary patients, a fitness programme engaging them in a 1-h daily walk resulted in significant decreases in systemic inflammation parameters.^142^ An important advantage of physical exercise is its potential to prevent metabolic side-effects of antipsychotics.^143^ Exercise also attenuates progressive grey matter loss in the early stages of schizophrenia^144^ and leads to an increase in hippocampal volume in patients.^145^ Whether exercise is effective in FEP or ARMS has not been tested, but may show particular promise in view of the absence of harmful side-effects.

Other non-pharmacological interventions consist of neuromodulation, using repetitive transcranial magnetic stimulation (rTMS) or transcranial direct current stimulation. Theoretically, these interventions can improve GABA-ergic inhibition with minimal side effects.^146,147^ Recent advances in spatial and temporal precision of these neuromodulation techniques allow for specific enhancement of neural synchrony in a particular brain area (for example, the dorsolateral prefrontal cortex), which can improve cognitive functions, such as working memory.^148,149^

### Towards personalized medicine for patients with schizophrenia

Schizophrenia most likely develops from several different mechanisms, among which are increased DA synthesis, NMDAr hypofunction and increased proinflammatory status of the brain. Neuroimaging techniques may help to tailor treatments to the needs of individual patients. Given that the vast majority of FEP patients respond well to an antipsychotic agent, it does not seem worthwhile to use invasive and expensive PET scans for selection before a first medication trial. In the FEP patients who fail to respond to a first antipsychotic trial, however, further investigations may be valuable.^112,113^ MRS can be performed on most clinical magnetic resonance imaging scanners. Both the peak in glx observed during ARMS and FEP and the subsequent decrease observed in more chronic stages of the illness could be targeted with glutamatergic drugs. Likewise, decreases in GABA could be compensated with selective GABA-agonists. Alternatively, hypofunction of the GABA-ergic interneurons could be compensated by increasing cortical inhibition with targeted neuromodulation.^147^ Increased proinflammatory status of the brain, in particular increased microglia cell activation, can be detected with PET scans using the PK11195 tracer, but this is an invasive and expensive technique. As increased proinflammatory status may not be restricted to the brain, but may be systemic in a subset of patients with schizophrenia,^49^ measurements of proinflammatory cytokines in peripheral blood, such as the IL-1 receptor antagonist, IL-6 and sIL-2R could provide a simple screening method to select patients for augmentation with antiinflammatory drugs.^150,151^ Another approach could be to measure the concentration of C-reactive protein, which is a general reflection of heightened (native and adaptive) immune activity,^152,153^ but also of metabolic syndrome, stress and even smoking.^154^

## Conclusion

At the time of first psychotic symptoms, neurobiological processes underlying schizophrenia have already been ongoing for many years. Although increased DA synthesis may be the final common pathway to psychosis, hypofunction of the NMDAr, associated decreased GABA-ergic signalling and increased proinflammatory status of the brain may be important mechanisms underlying cognitive dysfunction. The contribution of these pathophysiological pathways to the clinical picture of schizophrenia most likely varies per individual. If we aim to intervene before the window of opportunity is closed and deviations in the brain have become hard-wired, it will be key to include cognitive deterioration in the diagnosis of schizophrenia instead of postponing diagnosis until the onset of psychotic symptoms many years later. Meanwhile, effective interventions, with high numbers-needed-to-harm, should be considered for at-risk groups.

## Figures and Tables

**Table 1A:** Dopamine in ultra-high-risk subjects

*Study*	*Technique and ligand*	*Sample size*	*Main finding*
Allen *et al.*^155^	PET (18)F-DOPA	16 UHR-nt 5 UHR-t, 5 HC	Striatal DA synthesis capacity all UHR=HC, but increased in UHRt
Bloemen *et al.*^156^	[123I]-IBZM SPECT baseline and with α-methyl-para-tyrosine	14 UHR 15 HC	Postsynaptic DA: UHR=HC baseline and after DA depletion
Egerton *et al.*^157^	PET (18)F-DOPA	26 UHR 20 HC	Striatal DA synthesis capacity UHR>HC (ES 0.8)
Fusar-Poli *et al.*^158^	PET (18)F-DOPA	20 UHR, 14 HC^a^	Striatal DA synthesis capacity UHR>HC (ES 0.75)
Fusar-Poli *et al.*^159^	PET (18)F-DOPA	20 UHR, 14 HC^a^	Striatal DA synthesis capacity UHR>HC correlation left inferior frontal activation and striatal dopamine
Hirvonen *et al.*^160^	((11)C)-labelled raclopride PET	11 GHR (of which 5 MZ and 6 DZ unaffected co-twin), 7 HC	Striatal D2 MZ>DZ/HC
Howes *et al.*^161^	PET (18)F-DOPA	24 UHR^a^ 7 SCZ, 12 HC	Striatal DA synthesis capacity UHR>HC (ES 0.75) Schiz >>HC (ES 1.25)
Howes *et al.*^37^	PET (18)F-DOPA	20 UHR^a^ scanned twice	Striatal DA synthesis↑from UHR to FEP (ES 1.125 ) in 8 converters
Howes *et al.*^38^	PET (18)F-DOPA	30 UHR^a^ 29 HC	Striatal DA synthesis capacity UHR>HC, converters >non-converters
Suridjan *et al.*^162^	PET [11C]-(+)-PHNO	13 CHR, 13 FEP 12 HC	No difference in non-displaceable DA D2/D3 binding potential

Abbreviations: CHR, clinical high risk; DA, dopamine; ES, effect size; FEP, first-episode psychosis; PET, positron emission tomography; Schiz, patients with schizophrenia; UHR-nt, non-transition; UHR-t, transition to psychosis; UHRS, ultra-high-risk subjects.

a Samples overlap.

**Table 1B:** Dopamine in medication-free schizophrenia patients with a first psychotic episode

*Study*	*Technique and ligand*	*Sample size*	*Main finding*
Abi-Dargham *et al.*^163^	[^11^C]NNC 112 PET	16 SCZ of whom 7 FEP 16 HC	D1r bp DLPFC patients>HC
Abi-Dargham *et al.*^164^	[^11^C]NNC 112 PET	30 FEP 15 HC	DAT FEP<HC
Buchsbaum *et al.*^165^	(18)F-fallypride PET	15 FEP 15 HC	bp FEP<HC
Corripio *et al.*^166^	123I-IBZM SPECT	18 FEP^a^ 12 HC	D2r bp FEP>HC
Corripio *et al.*^167^	123I-IBZM SPECT	37 FEP^a^ 18 HC	D2r striatal/frontal ratios FEP>HC in those with SCZ
Glenthoj *et al.*^168^	123I-IBZM SPECT	25 FEP 20 HC	Extra striatal D2/D3 DAr bp FEP=HC
Graff-Guerrerro *et al.*^169^	[(11)C]-(+)-PHNO PET	13 FEP^a^ 13 HC	Nondisplaceable D2/D3 bp FEP=HC
Graff-Guerrerro *et al.*^170^	[(11)C]-(+)-PHNO PET	13 FEP^a^ 13 HC	D2/D3 bp FEP=HC
Hietala *et al.*^171^	[18F]-DOPA PET	7 FEP 8 HC	Striatal DA synthesis capacity FEP>HC
Hietala *et al.*^172^	[18F]-DOPA PET	10 FEP 10 HC	Striatal DA synthesis capacity FEP>HC
Hsiao *et al.*^173^	[99mTc]TRODAT SPECT	12 FEP 12 HC	DAT FEP=HC
Karlsson *et al.*^174^	[(11)C]SCH 23390 PET	10 FEP 10 HC	D1r bp FEP=HC
Laakso *et al.*^175^	[18F]CFT PET	9 FEP 9 HC	DAT FEP=HC
Lavalaye *et al.*^176^	[123I]FP-CIT SPECT	36 SCZ of whom 10 FEP 10 HC	DAT FEP=HC
Lehrer *et al.*^177^	(18)F-fallypride PET	33 SCZ of whom 14 FEP 18 HC	bp medial thalamus SCZ<HC (ES=0.89)
Lindstrom *et al.*^178^	[11C]-DOPA PET	12 SCZ of whom 10 FEP 10 HC	Striatal DA synthesis capacity FEP>HC
Mateos *et al.*^179^	[123I]FP-CIT SPECT	20 FEP 10 HC	DAT FEP<HC
Mateos *et al.*^180^	[123I]FP-CIT SPECT	30 FEP 15 HC	DAT FEP<HC
Mateos *et al.*^181^	[123I]FP-CIT SPECT	20 FEP 15 HC	DAT FEP<HC
Nozaki *et al.*^182^	[11C]-DOPA PET	18 SCZ of whom 14 FEP 10 HC	bp FEP>HC
Safont *et al.*^183^	(123)I-IBZM SPECT	37 FEP^a^ 18 HC	D2r bp cannabis users=non-users
Schmitt *et al.*^184^	([99mTc]TRODAT-1 SPECT	10 FEP 10 HC	DAT FEP=HC
Schmitt *et al.*^185^	([99mTc]TRODAT-1 SPECT	28 FEP 12 HC	DAT FEP=HC
Schmitt *et al.*^186^	[99mTc]TRODAT-1 and [123I]IBZM SPECT	20 FEP 12 HC	DAT FEP=HC D2r bp FEP=HC
Schmitt *et al.*^187^	123I-IBZM SPECT	23 FEP 10 HC	D2r bp FEP<HC
Schmitt *et al.*^188^	[99mTc]TRODAT-1 and [123I]IBZM SPECT	12 FEP 12 HC	DAT FEP>HC D2r bp FEP=HC
Talvik *et al.*^189^	[11C]FLB 457 PET	9 FEP 8 HC	D2/D3 bp right thalamus FEP<HC,
Talvik *et al.*^190^	[(11)C]raclopride PET	18 FEP 17 HC	D2 bp right thalamus FEP<HC,
Yang *et al.*^191^	[99mTc]TRODAT SPECTand ([(123)I]IBZM) SPECT	11 FEP 12 HC	DAT FEP=HC D2/D3 bp FEP=HC
Yasuno *et al.*^192^	[(11)C]FLB 457 PET	10 FEP 19 HC	D2 bp FEP<HC thalamus

Abbreviations: bp, binding potential; D1r, dopamine D1 receptor; D2r, dopamine D2 receptor; DAT, striatal dopamine transporter; ES, effect size; FEP, first-episode psychosis; PET, positron emission tomography; SCZ, schizophrenia.

a Samples overlap.

**Table 2A:** Glutamate and glutamine in ultra-high-risk subjects

*Study*	*Technique and area*	*Sample size*	*Main finding*
Bloemen *et al.*^193^	^1^H-MRS hippocampus	11 UHR 11 HC	glu UHR<HC (ES=0.22)
De la Fuente-Sandoval *et al.*^66^	^1^H-MRS dorsal-caudate cerebellum	18 UHR^a^ 18 medication-naive FEP 40 HC	Dorsal-caudate glu: UHR=FEP>HC cerebellar glu: UHR=FEP=HC
De la Fuente-Sandoval *et al.*^67^	^1^H-MRS dorsal-caudate nucleus	19 UHR^a^ (7 UHR-t) 26 HC	glu UHR-t>UHR-nt UHR-ts>HC (ES=1.39)
Fusar-Poli *et al.*^194^	^1^H-MRS thalamus, ACC, hippocampus	24 UHR^a^ 17 HC	glu thalamus UHR<HC
Keshavan *et al.*^65^	^1^H-MRS frontal, occipital, temporal, parietal, basal	40 GHR 46 HC	Inferior parietal/occipital region glx GHR>HC
Natsubori *et al.*^72^	^1^H-MRS medial prefrontal	24 UHR, 73 HC	glx UHR=HC
Purdon *et al.*^195^	1H-MRS medial frontal	15 GHR 14 HC	glx GHR=HC, but more variability in glx in GHR
Stone *et al.*^69^	^1^H-MRS, thalamus ACC, hippocampus	27 UHR^a^ 27 HC	glu thalamus UHR<HC gln ACC UHR>HC
Tandon *et al.*^68^	^1^H-MRS thalamus caudate ACC	23 GHR 24 HC	glx thalamus and caudate GHR>HC, ACC glx HR=HC
Tibbo *et al.*^64^	^1^H-MRS right medial frontal	20 GHR 22 HC	glx GHR>HC
Valli *et al.*^196^	^1^H-MRS medial temporal, ACC, thalamus	22 UHR 14 HC	glu UHR=HC (trend in thalamus: UHR<HC)
Yoo *et al.*^71^	^1^H-MRS ACC, DLPFC, thalamus	22 GHR 22 HC	glx HR=HC

Abbreviations: ACC, anterior cingulate gyrus; ES, effect size; FEP, first-episode psychosis; GHR, genetic high risk; gln, glutamine; glu, glutamate; glx, glutamate+glutamine; MRS, magnetic resonance spectroscopy; UHR, ultra-high-risk subjects, UHR-nt, non-transition; UHR-t, transition to psychosis.

a Samples overlap.

**Table 2B:** Glutamate and glutamine in first-episode psychosis subjects

*Study*	*Technique and area*	*Sample size*	*Main finding*
Bartha *et al.*^73^	^1^H-MRS medial prefrontal	14 FEP 10 HC	glu prefrontal FEP>HC
Bartha *et al.*^197^	^1^H-MRS medial temporal	11 FEP 11 HC	glx FEP=HC
Bustillo *et al.*^75^	^1^H-MRS AC, frontal white, thalamus	14 FEP 10 HC	gln/glu ratio AC FEP>HC
Bustillo *et al.*^79^	^1^H-MRS 1 slice parallel to AC-PC above ventricles	30 Medicated FEP 28 HC	glx medicated FEP=HC
De la Fuente-Sandoval *et al.*^66^	^1^H-MRS precommissural dorsal-caudate cerebellar cortex	18 FEP 40 HC	glu precommissural dorsal-caudate FEP>HC glu cerebellar cortex FEP=HC
De la Fuente-Sandoval *et al.*^198^	^1^H-MRS striatal cerebellum	24 Medication-naive FEP, 18 HC Scanned twice	Striatal glu: FEP>HC cerebellar glu: FEP>HC after 4 weeks medication: glu FEP= glu HC
Galinska *et al.*^78^	^1^H-MRS frontal, temporal, thalamus	30 Medicated FEP, 19 HC	glx medicated FEP=HC
Natsubori *et al.*^72^	^1^H-MRS medial prefrontal	19 FEP, 73 HC, 25 ChSz	glx FEP=HC ChSz< HC
Ohrmann *et al.*^199^	^1^H-MRS DLPFC	18 FEP, 21 HC, 21 ChSz	glx FEP=HC, ChSz< HC FEP
Ohrmann *et al.*^200^	^1^H-MRS DLPFC	18 FEP, 20 HC	glx FEP=HC
Olbrich *et al.*^201^	^1^H-MRS DLPFC hippocampus	9 Medicated FEP 32 HC	Thalamus glu FEP>HC hippocampus same trend
Stanley *et al.*^76^	^1^H-MRS DLPFC	10 Medicated FEP, 11 FEP, 24 HC	glu FEP>HC (trend) gln FEP=HC
Théberge *et al.*^202^	^1^H-MRS ACC thalamus	21 FEP 21 HC	gln thalamus and ACC FEP>HC
Théberge *et al.*^203^	^1^H-MRS ACC thalamus	21 FEP 21 HC	gln thalamus and ACC FEP>HC
Wood *et al.*^77^	^1^H-MRS temporal	15 FEP, 19 HC 19 medicated FEP,	glx FEP=HC
Wood *et al.*^204^	^1^H-MRS medial temporal	34 FEP (15 medication-naive), 19 HC	glx FEP=HC

Abbreviations: ChSz, chronic schizophrenia patients; DLPFC, dorsolateral prefrontal cortex; FEP, first-episode psychosis, FEP patients are medication free unless defined otherwise; gln, glutamine; glu, glutamate; glx, glutamate+glutamine; nt, non-transition; t, transition to psychosis.

**Table 3:** Markers of low-grade inflammation in the brain of patients with schizophrenia

*Study*	*Technique and ligand*	*Sample*	*Main finding*
Arnold *et al.*^205^	Microglial infiltrates in postmortem brains	23 SCZ 14 HC	No difference
Bayer *et al.*^206^	Microglial activation in postmortem brains	14 SCZ 13 HC	3 SCZ patients with abundant activated microglia density
Bruton *et al.*^207^	Neuropathological examination	56 SCZ 56 HC	More fibrillary gliosis than HC
Busse *et al.*^208^	HLA-DR+ microglial cells in postmortem brains	17 SVZ 11 HC	Microglia activation increased, especially in paranoid group
Doorduin *et al.*^97^	PET PK11195	7 SCZ 8 HC	More activated microglia in SCZ
Falke *et al.*^209^	Microgliosis	11 SCZ 11 HC	No difference
Fillman *et al.*^210^	mRNA expression levels in postmortem brains	20 SCZ 20 HC	40% SCZ: increased microglia density and proinflammatory pathways
Fisman^211^	Neuropathological examination	8 SCZ 10 HC	Microglial nodules in 5 SCZ and 0 HC
Kurumaji *et al.*^212^	PK11195 in postmortem brains	13 SCZ 10 HC	Decrease/no difference in SCZ
Nasrallah *et al.*^213^	Glial counting in corpus callosum	18 SCZ 10 HC	Increased gliosis in SCZ
Radewycz *et al.*^214^	HLA-DR+ microglial numerical density	7 SCZ 10 HC	Increased density of activated microglia in temporal and frontal cortex
Rao *et al.*^215^	Microglial marker CD11b in postmortem brains	10 SCZ 10 HC	Increased microglia activation in SZ
Roberts *et al.*^216^	Antibody to glial fibrillary acidic protein	5 SCZ 7 HC	No difference in gliosis
Roberts *et al.*^217^	Antibody to glial fibrillary acidic protein	18 SCZ 12 HC	No difference in gliosis
Steiner *et al.*^218^	HLA-DR on microglia in postmortem brains	16 HC 16 SCZ	No difference
Steiner *et al.*^219^	HLA-DR+ microglial numerical density	16 SCZ 10 HC	No difference in microglia cell density
Steiner *et al.*^220^	Microglial HLA-DR expression in postmortem brains	16 SCZ 10 HC	No general difference, increased in suicidal (=younger) SCZ patients
Stevens *et al.*^221^	Neuropathological examination	28 SCZ 16 HC	Gliosis in 16 SCZ and in 1 HC
Stevens *et al.*^222^	Postmortem neuropathological examination	5 SCZ 7 HC	No difference in gliosis
Togo *et al.*^223^	Expression of CD40 in postmortem brains	4 SCZ 2 HC	Increased microglia activation
Van Berckel *et al.*^96^	PET PK11195	10 SCZ 10 HC	More activated microglia in SCZ
Wierzba Bobrowic *et al.*^224^	MHC II on microglial cells in postmortem brains	12 SCZ	Degeneration of activated microgial cells
Wierzba Bobrowic *et al.*^225^	MHC II on microglial cells in postmortem brains	9 SCZ 6 HC	More activated microglia cells in SCZ

Abbreviations: HC, healthy controls; PET, positron emission tomography; SCZ, patients with schizophrenia.
